# Parental Emotion Socialization and Difficulties in Emotion Regulation in Adolescents: A Network Analysis

**DOI:** 10.1007/s10964-025-02222-8

**Published:** 2025-07-30

**Authors:** Márcia Silva Azevedo, Liliana Meira, Daniela Pascoal Câmara, Tiago Bento Ferreira, Eva Costa Martins

**Affiliations:** 1 Department of Social and Behavioural Sciences, University of Maia – UMAIA, Maia, Portugal; 2 Center for Psychology at University of Porto – CPUP, Porto, Portugal

**Keywords:** Parental emotion socialization, Emotion regulation, Adolescence, Network analysis

## Abstract

Parental emotion socialization strategies influence adolescent emotion regulation development. However, there are still doubts about the supportive/unsupportive role of each strategy. This may be likely due to limitations of prior research that analyzed these strategies in isolation, without considering their interdependence. This study explored interactive patterns among parental strategies, clarifying which combinations were associated with fewer emotion regulation difficulties. Participants were 503 adolescents (60.8% female; *M*_*age*_ = 14.54; *SD*_*age*_ = 1.17). Network and Expected Symptom Activity analyses indicated that *reward* and *override* strategies were associated with fewer emotion regulation difficulties, while *punishment*, *neglect*, and *magnification* were associated with greater difficulties. High *reward* (but not high *override*) mitigated emotion regulation difficulties unless alongside high *neglect* or two unsupportive strategies. *Override* helped buffered *neglect*’s negative effects when combined with high *reward*. High values in all strategies were associated with more emotion regulation difficulties, while low values with fewer. These results emphasize the interactive nature of parental emotional responses during adolescence, suggesting that no strategy is inherently adaptive or maladaptive—it depends on the context of co-occurring parental behaviors. The findings also support the “good enough” parenting perspective, showing that positive and negative behaviors can coexist and still be associated with fewer difficulties in emotion regulation. In this context, neglect appears to be more detrimental than other emotion socialization strategies.

## Introduction

Parental emotion socialization is a multifaceted process in which adolescents learn to understand, experience, express, and regulate emotions based on their parents’ reactions/responses (Eisenberg, 2020; Eisenberg et al., 1998). These parental emotion socialization practices have implications for the development of adolescents’ emotion regulation as they influence the way adolescents learn to express and regulate emotions, impacting their overall psychological adjustment (Breaux et al., 2022). However, prior research designs do not consider the interdependencies between these socialization responses, which does not clarify the quality of the emotion socialization strategies (i.e., supportive/unsupportive role). This study explored a new approach to assess the interactive patterns among parental strategies, clarifying which combinations were associated with fewer emotion regulation difficulties.

Adolescence is a critical period for the development of emotional and social competencies essential to long-term mental health (World Health Organization, 2024). In particular, middle adolescence (roughly 14–17 years) is marked by emotional reactivity and heightened sensitivity to social environments, which make adolescents especially susceptible to both risk and protective factors. In middle adolescence youth begin to express greater autonomy in managing emotions and interpersonal exchanges. However, they continue to rely on parental support and guidance, making parental emotion socialization relevant during this period (Morris et al., 2007). This stage differs from early adolescence (10–12/13 years), when puberty brings about rapid emotional changes and emotion regulation skills are still emerging, and late adolescence (18–21 years), characterized by greater independence and emotional maturity and less parental involvement (Salmela-Aro, 2011).

Parental Emotional socialization includes how parents express their emotions, the different emotional regulation strategies they use, their responses to children’s emotional expression, and how they teach and discuss emotions with children (Eisenberg et al., 1998). Affect theory (Tomkins, 1963) focuses on how socioemotional dispositions are transmitted from one generation to the next. Furthermore, emotional experiences in response to others or the environment (e.g. parental responsiveness or non-responsiveness) are internalized as emotional biases, or “ideoaffective organizations”. Thus, parental emotion socialization is shaped by parents’ affect ideologies, specifically their beliefs about which emotions are acceptable and how they should be regulated. Building on this theoretical work (Tomkins, 1963), research conceptualized *reward, punishment, neglect, override*, and *magnification* as emotion socialization strategies that are often used by parents to socialize/respond to their children’s negative emotions (Eisenberg et al., 1998). These parents’ responses can be supportive or unsupportive, with the supportive responses promoting adolescents’ emotion regulation development and the unsupportive increasing the difficulties in emotion regulation. This, in turn, impacts youth ability to regulate negative emotions and leads to detrimental consequences for their psychological adjustment and the onset of psychopathological symptoms (McNeil & Zeman, 2021).

*Reward* (providing comfort, empathizing and problem solving) has consistently been classified as a supportive strategy (McNeil & Zeman, 2021). Strategies like *punishment* (expressing disapproval for the expressed emotions), *neglect* (appearing unavailable or ignoring the adolescent’s emotional expression), *override* (silence/downplaying the expression of the emotion through dismissing or distracting behavior), and *magnification* (responding with the same emotion with equal or stronger intensity) have been considered unsupportive strategies (McCord & Raval, 2016). However, literature shows that there are inconsistencies regarding the unsupportive role of the latter four strategies (Guo et al., 2017; Hu et al., 2023). The main inconsistencies in literature concern the override strategy. Although often conceptualized as an unsupportive or dismissive response (McCord & Raval, 2016; McNeil & Zeman, 2021), some studies have identified override as supportive, suggesting that distracting adolescents from their emotions may enhance their ability to use strategies to regulate emotions efficiently (O’Neal & Magai, 2005; Silk et al., 2011). Findings regarding magnify are generally more consistent with an unsupportive role, as it can intensify negative emotions and lead to greater emotion dysregulation (O’Neal & Magai, 2005; Silk et al., 2011). However, its impact may vary depending on the emotion, serving a supportive role for sadness or fear, but not for anger (Garside & Klimes-Dougan, 2002). By contrast, punishment and neglect tend to exhibit a more consistent pattern of an unsupportive response (Guo et al., 2017).

Some contradictory findings may be due to the use of what has been referred to as a variable-centered approach to research, in which different parental emotion socialization responses are assessed in isolation (McKee et al., 2022). This research design does not consider the interdependencies between these socialization responses (Wang et al., 2019) and how these interrelations impact youth developmental outcomes (McKee et al., 2022). This is a limitation, given that parents implement various strategies to respond to adolescents’ emotions simultaneously or in succession, and one behavior may have a different meaning depending on the presence of other behaviors. Therefore, recent research has shifted to what has been referred to as a person-centered approach by analyzing potential profiles/patterns of parents’ responses to adolescents’ emotions. Some studies identified four profiles, namely, coaching/accepting (above average scores on all the supportive responses and below average for non-supportive responses), blended (average level of both supportive and non-supportive responses), punishing/minimizing (below average supportive and above average non-supportive responses), and low-involvement (below average in all responses) (Howe & Zimmer-Gembeck, 2022, 2023; McKee et al., 2022; Wang et al., 2019). Another study identified two profiles, namely, high-involvement profile (higher scores on supportive and unsupportive responses) and a low-involvement profile (lower scores on supportive and unsupportive responses, Miller et al., 2015). Coaching/accepting and blended profiles have been generally associated with healthier youth development (i.e., better emotion regulation and fewer internalizing and externalizing symptoms), in contrast with punishing/minimizing, low involvement, and high involvement profiles (i.e., profiles associated with children with less emotion regulation and more internalizing and externalizing symptoms) (Howe & Zimmer-Gembeck, 2022; McKee et al., 2022; Wang et al., 2019).

However, as with variable-centered studies, there are inconsistencies regarding the adaptative/maladaptive role of these parents’ profiles. For example, children of parents with a low involvement profile were found to have better emotion regulation than those of parents with a coaching/accepting profile (Howe & Zimmer-Gembeck, 2023). Moreover, this person-centered approach again does not clarify the quality of the individual emotion socialization strategies because the profile only captures a static configuration of the associations between them. By not capturing the dynamics of the interactions between emotion socialization strategies and how these interactions are associated with youth developmental outcomes, the question about the association of each one remains unanswered.

It may help to consider that parental emotion socialization strategies have emergent properties rather than intrinsic ones so that their adaptive/maladaptive nature does not inherently belong to the strategy itself, but emerges from a complex system of interactions and relationships between the different emotion socialization strategies. Therefore, the nature of each individual strategy will only be apparent in the context of dynamic patterns between these strategies. For this to be accomplished, a different methodological approach may be necessary. A new approach to studying emotion socialization may be based on psychological networks. These are conceptualized as complex systems where psychological phenomena emerge from the result of the relations between the network’s components (Borsboom, 2017; Borsboom & Cramer, 2013). Unlike traditional approaches, psychological networks are conceptualized as systems of directly and causally connected variables, rather than as effects of a single latent construct (Borsboom & Cramer, 2013; Epskamp & Fried, 2018). In contrast, moderation analyses assess predefined linear interactions between variables (Hayes, 2013), and latent class analysis identifies unobserved subgroups / profiles within a population (Spurk et al., 2020), both of which may overlook the complex, dynamic interrelations captured by network models.), both of which may overlook the complex, dynamic interrelations captured by network models.

A psychological network is constituted of variables (represented by nodes) that are connected through relations (represented by edges; Borsboom & Cramer, 2013). Networks are dynamic models of phenomena and display nontrivial connectivity and activation patterns. The edges’ weight, type, and directionality indicate the connectivity between variables. Connectivity and activation patterns are intertwined since the activation of one variable will lead to the activation of other variables to which it is strongly connected (i.e., the activation of one emotion socialization strategy leads to the activation of another emotion socialization strategy to which it is strongly connected, Borsboom, 2017).

One way to analyze the activation patterns is by using Expected Symptom Activity (Lunansky et al., 2021). Expected Symptom Activity is a statistical method that estimates the impact of external factors on the activation of the networks’ variables based on the existing connections between those variables. Therefore, this research focus on the application of this innovative method to study a network comprised of emotion socialization strategies (i.e., reward, punishment, neglect, override, and magnify) and emotion regulation difficulties (i.e., difficulties in access to emotion regulation strategies, non-acceptance of emotion responses, lack of emotion awareness, impulse control difficulties, difficulties in acting by goals, and lack of emotion clarity). Emotion dysregulation as the chosen outcome measure, since there is an established link between parental emotion socialization and emotion regulation (Breaux et al., 2022; Eisenberg, 2020; Eisenberg et al., 1998).

## Current Study

Previous research on parental emotion socialization has mainly analyzed these strategies in isolation, without considering their interdependence. This study applied Expected Symptom Activity to describe the interactive and dynamic patterns between parental emotion socialization strategies in response to adolescent’s negative emotions. This approach intended to clarify which combinations are associated with fewer adolescents’ emotion regulation difficulties and, ultimately, the supportive or unsupportive quality of each emotion socialization strategy in the presence of other parenting strategies. The first aim was to describe the straightforward relation of each emotion socialization strategy with the difficulties in emotion regulation by maintaining the other emotion socialization strategies at a low level. The second aim was to clarify the impact of different configurations (i.e., profiles identified in the person-centered approach) of emotion socialization strategies on the difficulties in emotion regulation. The third aim was to explore different/new network configurations among parent emotion socialization strategies and determine on which conditions the supportive/unsupportive quality of an individual emotion socialization strategy emerges or is ceased by the effect of high values of another or the remaining emotion socialization strategies.

## Method

### Participants

A total of 503 participants (60.8*%* female) were recruited by convenience sampling from four middle and high schools in the island of São Miguel, Autonomous Region of the Azores, Portugal. Participants aged between 13 and 17 years old (*M* = 14.54; *SD* = 1.17) were included. Adolescents identified with cognitive impairment by the school services were excluded from the sample. The final sample included adolescents attending the 8th grade (*n* = 142; 28.2*%*), 9th grade (*n* = 120; 23.9*%*), 10th grade (*n* = 130; 25.8*%*) and 11th grade (*n* = 111; 22.1*%*).

### Measures

A sociodemographic questionnaire was used to gather information about the age, gender, and education level of adolescents.

#### Emotion Socialization

Emotion Socialization Scale (ESS; Magai & O’Neal, 1997; Portuguese version Costa Martins et al., 2018) is a youth self-report questionnaire developed from its inception to assess youth’s perception about five parental emotion socialization strategies – reward, punishment, neglect, override, and magnify – regarding four emotions, three negative – sadness, anger, fear – and one positive – overjoy (Magai & O’Neal, 1997). This instrument has supportive evidence of construct validity in the original and Portuguese adaptation both with adolescents’ samples and evidence of convergent validity (original sample, relates to internalizing and externalizing behaviors, O’Neal & Magai, 2005; Portuguese sample, relates to maternal rearing practices namely, emotional warmth, rejection and overprotection, Costa Martins et al., 2018).

Adolescents rated how much their parents were likely to react to each emotion by using a variety of socializing strategies (60 items; 15 items per emotion, three items per emotion socialization strategy) on a 5-point Likert scale (1 = *never*, and 5 = *very often*). Since only negative emotions will be used, the negative emotions subscale scores (e.g., reward of sadness, reward of anger, and reward of fear) were averaged to obtain each socialization strategy (e.g., reward) in accordance with the Portuguese factorial structure. Examples of items of socialization strategies (e.g., for anger) include: Reward (e.g., “When I was angry, my mother/father/caregiver helped me deal with the issue that made me angry.”), Override (e.g., “When I was angry, my mother/father/caregiver bought me something I liked.”), Punishment of anger (e.g., “When I was angry, my mother/father/caregiver told me that I was acting too childish for my age.”), Neglect (e.g., “When I was angry, my mother/father/caregiver gave me attention. (reverse score)”), and Magnify (e.g., “When I was angry, my mother/father/caregiver expressed that they were very angry.”).

In the present study, internal consistency using Cronbach’s alpha and McDonald’s omega for the five subscales reached good values ranging from 0.751–0.914, and 0.743–0.937, respectively (Kalkbrenner, 2023; Table 1).

**Table 1:** Descriptive statistics and internal consistency for the five negative emotions socialization strategies and for the difficulties in emotion regulation (*N* = 503)

	*Min*	*Max*	*M (SD)*	Internal Consistency	
				*α* [95*%IC BCa*]	Ω [95*%IC BCa*]
Emotion Socialization Strategies					
Reward	3	15	11.2 (2.9)	0.936 [0.925, 0.946]	0.937 [0.926, 0.946]
Punishment	3	13.3	7.1 (2)	0.751 [0.714, 0.784]	0.743 [0.671, 0.784]
Neglect	3	15	7.1 (2.7)	0.914 [0.901, 0.926]	0.919 [0.906, 0.931]
Override	3	14.7	9.1 (2.3)	0.845 [0.820, 0.867]	0.847 [0.821, 0.869]
Magnify	3	15	6.3 (2)	0.809 [0.779, 0.838]	0.809 [0.778, 0.838]
Difficulties in Emotion Regulation					
Strategies	8	39	19.8 (7)	0.854 [0.833, 0.873]	0.858 [0.836, 0.876]
Nonacceptance	6	30	14.9 (6.6)	0.908 [0.892, 0.921]	0.910 [0.894, 0.923]
Awareness	6	30	16 (4.8)	0.773 [0.738, 0.803]	0.782 [0.749, 0.811]
Impulse	6	30	15.7 (6.1)	0.878 [0.860, 0.894]	0.891 [0.875, 0.905]
Goals	5	25	16.4 (4.8)	0.841 [0.814, 0.865]	0.846 [0.819, 0.868]
Clarity	5	25	12.6 (4.5)	0.810 [0.779, 0.839]	0.811 [0.778, 0.838]
Total	36	165	95.5 (24.1)	0.930 [0.921, 0.939]	0.931 [0.921, 0.941]

*Min* minimum, *Max* maximum, *M* mean, *DP* standard deviation, *α* cronbach alpha, *Ω* mcdonald omega, 95*%IC BCa* bias corrected and accelerated confidence intervals, *Strategies* limited access to emotion regulation strategies, *Nonacceptance* nonacceptance of emotional responses, *Awareness* lack of emotional awareness, *impulse* impulse control difficulties, *Goals* difficulties engaging in goal directed behavior, *Clarity* lack of emotional clarity

#### Emotion Regulation

Difficulties in Emotion Regulation Scale (DERS; Gratz & Roemer, 2004; Portuguese version Coutinho et al., 2010) is a self-report questionnaire used to assess six adolescent difficulties in the process of emotion regulation, namely: (1) limited access to emotion regulation strategies (e.g., “When I’m upset, I believe that there is nothing I can do to make myself feel better.”) ; (2) nonacceptance of emotion responses (e.g., “When I’m upset, I feel guilty for feeling that way.”); (3) lack of emotion awareness (e.g.,” I pay attention to how I feel. (reverse score)”); (4) impulse control difficulties (e.g., “When I’m upset, I lose control over my behaviors.”); (5) difficulties in acting in accordance with goals (e.g., “When I’m upset, I have difficulty getting work done.”); and (6) lack of emotion clarity (e.g., “I have difficulty making sense out of my feelings.”). Participants indicate how often each item applies to themselves (36 items) on a 5-point Likert scale [1 = *almost neve*r (0–10*%*) and 5 = *almost always* (91–100*%*)]. Higher scores indicate greater difficulties in emotion regulation (i.e., greater emotion dysregulation). In the present study, internal consistency using Cronbach’s alpha and McDonald’s omega for the total scale and the six subscales reached good values ranging from 0.773–0.930, and 0.782–0.931, respectively (Kalkbrenner, 2023; Table 1). This questionnaire was originally developed for adult respondents but have successfully been used in adolescents’ samples either in its original language (Neumann et al., 2010) or with Portuguese adolescents (Sousa et al., 2023). It also shows convergent validity (original sample, relates to externalizing and internalizing problems, Neumann et al., 2010; Portuguese sample, relates to depression, anxiety, and stress symptomatology, positive and negative affect, and cognitive reappraisal and expressive suppression, Sousa et al., 2023).

### Procedures

The research project was approved by the Council of Ethics and Deontology of the University of Maia (n° 100/2022). Subsequently, the data collection was authorized by the administration of each of the four schools (i.e., Presidents of the Executive Board). The study was then presented to the adolescents within the school setting, with the help of the teacher in the classroom. In the first session, the objectives of the study and the Informed Consent (IC) were presented. In a second session, the ICs signed by parents and adolescents were collected, and the questionnaires were administered, ensuring the anonymity of the data. Only the adolescents whose parents signed the IC were allowed to participate. Data collection was carried out by a Master’s student in Clinical and Health Psychology under the supervision of the person responsible for the study.

### Data Analysis

For data analysis, the R version 4.1.3 program was used (R Core Team, 2022). Descriptive statistics were carried out with the *summarytools* R package version 1.0.1 (Comtois, 2022). There were no missing values.

#### Expected Symptom Activity Analysis Applied to Difficulties in Emotion Regulation

Expected Symptom Activity (Lunansky et al., 2021) was carried out to analyze how increasing or decreasing the emotion socialization strategies influence the activity of the difficulties in emotion regulation. First, a network model of the associations between emotion socialization strategies (i.e., reward, punishment, neglect, override, and magnify) and emotion regulation difficulties (i.e., difficulties in access to emotion regulation strategies, non-acceptance of emotion responses, lack of emotion awareness, impulse control difficulties, difficulties in acting in accordance with goals, and lack of emotion clarity), was estimated. A Mixed Graphical Model (MGM; *bootnet* with *mgm* default; Epskamp et al., 2018; Haslbeck & Waldorp, 2020) was estimated using the *bootnet* R package version 1.5.5 (Epskamp & Fried, 2023). A 10-fold cross-validation was used to select the regularization parameter. Nodes represent the study variables, edges represent partial correlation coefficients between two nodes and the thickness of each edge indicates the edge-weight. Network stability was estimated using the *correlation stability (CS) coefficient* reaching an adequate value (see Supplementary Materials). The CS - coefficient should preferably be above 0.5, as recommended (Epskamp et al., 2018).

Centrality indices were measured with strength to assess the nodes’ importance and capacity to influence other nodes and the overall network. Strength measures how strongly a node is directly connected to other nodes and is computed as the sum of the absolute partial correlation coefficients of a node with the other nodes (Epskamp et al., 2018). The *qgraph* R package version 1.9.2 (Epskamp et al., 2012, 2023) was used to visualize the estimated network. Network stability was assessed using a bootstrapping method, which re-estimates the network through data resampling. The representation of robustness and stability of the network is presented in the Supplementary Materials (Figures S1–S3).

Afterward, the mean of the difficulties in emotion regulation was determined to estimate the Baseline Symptom Activity. Using Expected Symptom Activity, the five emotion socialization strategies were conditioned with their minimum score (i.e., 3) as well as their maximum score (i.e., 15), creating thirty-two possible simulations.

Specifically, the first aim was based on a variable-centered approach but was expanded by the network dynamic pattern. Nevertheless, the individual emotion socialization strategy was assessed as an emergent result of the interaction patterns in the network. Accordingly, one emotion socialization strategy at a time was conditioned with their maximum score and the other four strategies with their minimum score (e.g., the impact of high reward on network connectivity, when the other four strategies are low).

The second aim was based on a person-centered approach but was expanded by the network dynamic pattern. It followed the make-up of general profiles identified in the person-centered approach that combine high and low levels of different emotion socialization strategies (coaching/accepting; punishing/minimizing; low involvement, high involvement). The results of the first aim, when devising the parents’ patterns (i.e., emotion socialization strategies that were identified as adaptive would be included in purported “good” profiles), were also taken into consideration. Accordingly, reward and override strategies were conditioned with their maximum score and the other three strategies (i.e., punishment, neglect, and magnify) with their minimum score (coaching/accepting profile parents). Complementarily, punishment, neglect, and magnify strategies with their maximum score and the other two strategies (i.e., reward and override) with their minimum score (punishing/minimizing profile parents). Also, the five strategies were conditioned with their minimum score (low-involvement profile parents), and with their maximum score (high-involvement profile parents).

The third aim was based on a network approach to scrutinize the emergent properties of emotion socialization strategies by focusing on network configurations that were not analyzed in previous studies. Accordingly, first reward was conditioned with their maximum score and override with their minimum score and conditioned alternately the other three emotion socialization strategies with their minimum, as well as with their maximum score. Second, reward was conditioned with their minimum score and override with their maximum score and conditioned alternately the other three emotion socialization strategies with their minimum, as well as with their maximum score. Third, reward and override were conditioned with their maximum score and conditioned alternately the other three emotion socialization strategies with their minimum, as well as with their maximum score. Finally, reward and override were conditioned with their minimum score and conditioned alternately the other three emotion socialization strategies with their minimum, as well as with their maximum score. Following this, the Expected Symptom Activity was estimated as the sum of the resulting means of the emotion regulation difficulties.

For additional information on data analysis consult Supplementary Materials. R-script for these analyses, can be found here: https://osf.io/t2m5e/.

## Results

### Network Analysis

**Fig. 1:**
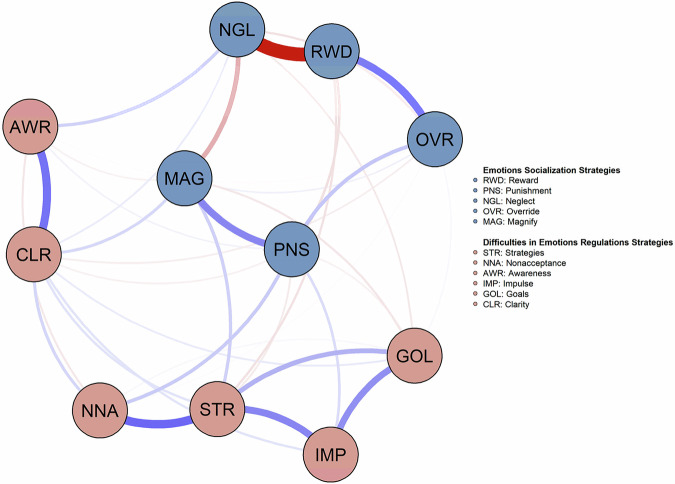
Emotion Socializations Strategies and Difficulties in Emotion Regulation Network. Estimated network structure between Emotion Socializations Strategies (blue nodes) and Difficulties in Emotion Regulation (pink nodes), based on a sample of 503 adolescents. with the Mixed Graphical Model (MGM). Blue edges (lines) represent positive associations and red edges (lines) represent negative associations. The thickness of the edges and color intensity represents the strength of the edges

The estimated network structure between emotions socialization strategies and difficulties in emotion regulation is presented in Fig. 1. Among the five emotion socialization strategies, the strongest connection weights were between reward and neglect (−0.776), followed by reward and override (0.401), punishment and magnify (0.370), neglect and magnify (−0.238), and punishment and override (0.178). Among the six difficulties in emotion regulation, the strongest connection weights were between limited access to emotion regulation strategies and nonacceptance of emotion responses (0.462), lack of emotion awareness and lack of emotion clarity (0.437), limited access to emotion regulation strategies and impulse control difficulties (0.370), impulse control difficulties and difficulties in acting in accordance with goals (0.339), limited access to emotion regulation strategies and difficulties in acting in accordance with goals (0.233) and nonacceptance of emotion responses and lack of emotion clarity (0.126). Furthermore, the strongest weights between the five emotions socialization strategies and the six difficulties in emotion regulation were between punishment and nonacceptance of emotion responses (0.161), magnify and limited access to emotion regulation strategies (0.151), neglect and lack of emotion awareness (0.145), magnify and lack of emotion clarity (0.129) and punishment and impulse control difficulties (0.108). All the network weights are shown in the Supplementary Materials (Table S1).

**Fig. 2:**
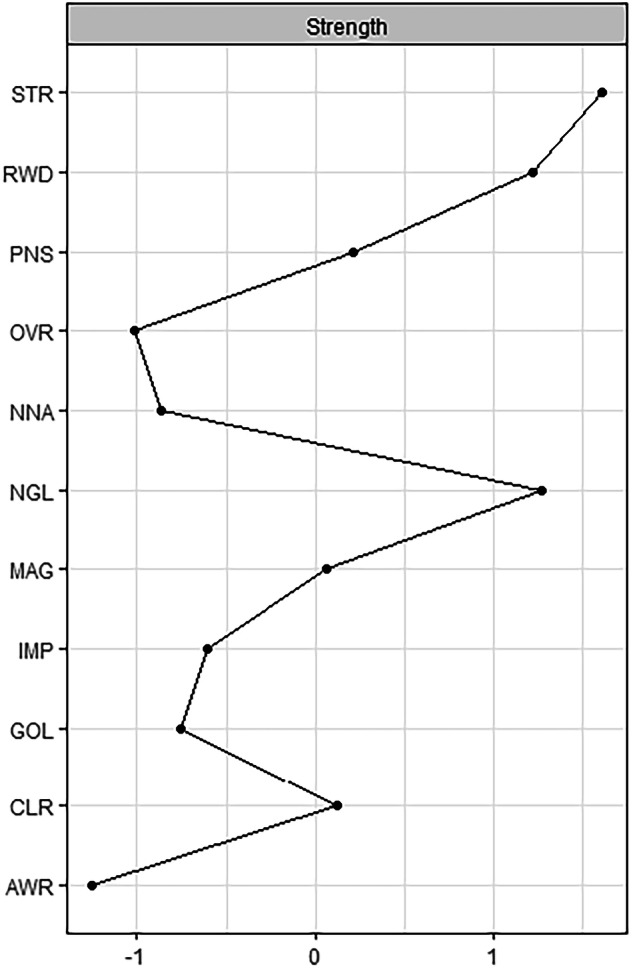
Centrality Plot. Centrality indices for the nodes of the present network are shown as standardized *z*-scores. The full names of the abbreviations can be found in Fig. 1

The network density of the edges between nodes was 0.141. The centrality indices estimated are shown in Fig. 2. Limited access to emotion regulation strategies (i.e., STR) had the highest strength in the network, followed by reward and neglect, suggesting their higher importance and their capacity to influence another node in the network. All centrality indices values are shown in the Supplementary Materials (Table S2).

### Expected Symptom Activity Analysis Applied to Difficulties in Emotion Regulation

Expected Symptom Activity for the conditioned emotion socialization strategies is presented in Table 2. The baseline (i.e., Baseline Symptom Activity) estimated was 95.53. Some of the different simulations decrease Expected Symptom Activity value in comparison with the baseline. In doing so, it generates changes in network activity, decreasing its connectivity (i.e., emotion socialization strategies are associated with the decreasing of the difficulties in emotion regulation). Other simulations increase Expected Symptom Activity value in comparison with the baseline, generating changes in the network activity, increasing its connectivity (i.e., emotion socialization strategies are associated with the increase of the difficulties in emotion regulation).

**Table 2:**
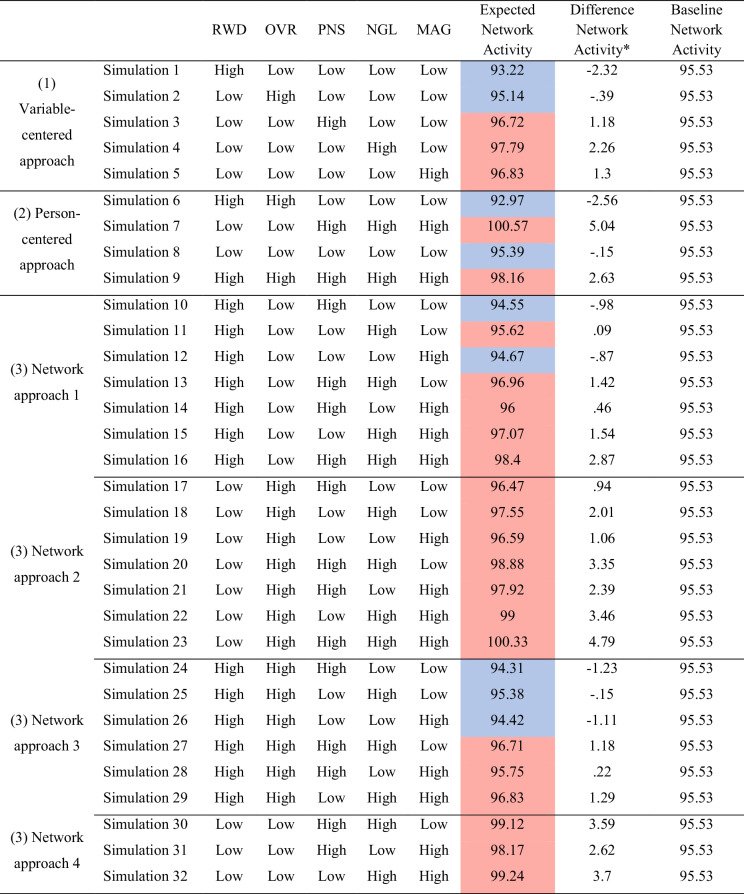
Expected Network Activity in Emotions Socialization Strategies

Red color represents higher values and blue color represents lower values, in comparation with the Baseline Network Activity (95.53)

*Difference Network Activity: difference between Baseline Network Activity and Expected Network Activity

#### Variable-Centered Approach Expanded by a Network Dynamic Pattern

The variable-centered approach expanded aim is presented in simulation 1–5, in which one emotion socialization strategy was conditioned with their maximum score at a time and the other four strategies with their minimum score. Expected Symptom Activity decreases in comparison with baseline, in simulation 1 and 2 (reward or override maximum score). High reward and high override were each associated with decreased network connectivity (less difficulties in emotion regulation) in the presence of the other low strategies. On the other hand, Expected Symptom Activity increases in comparison with baseline, in simulations 3–5 (punishment, neglect or magnify maximum score). High punishment, high neglect, and high magnify were associated with increased network connectivity (more difficulties in emotion regulation) in the presence of the other low strategies.

#### Person-Centered Approach Expanded by a Network Dynamic Pattern

The person-centered approach expanded aim is presented in simulation 6–9. In simulation 6, reward and override strategies were conditioned with their maximum score and the other three strategies with their minimum score. Expected Symptom Activity decreases in comparison with baseline. In this supportive profile (coaching/accepting), characterized by the presence in the network of high values of reward simultaneously with high levels of override and low levels of the other three strategies was associated with decreased network connectivity (less difficulties in emotion regulation). Also, punishment, neglect, and magnify strategies were conditioned with their maximum score and the other two strategies with their minimum score (simulation 7). Expected Symptom Activity increases in comparison with baseline. In this unsupportive profile (punishing/minimizing), characterized by the presence in the network of high values of punishment simultaneously with high levels of neglect, high levels of magnify, and low levels of the other two strategies, was associated with increased network connectivity (more difficulties in emotion regulation). These simulations showed the biggest difference from the baseline (simulation 6 = −2.56; simulation 7 = 5.04).

In simulation 8, the five strategies were conditioned with their minimum score. Expected Symptom Activity decreases in comparison with baseline. This low-involvement profile, characterized by the presence in the network of low values of all strategies, was associated with decreased network connectivity (less difficulties in emotion regulation). Also, the five strategies were conditioned with their maximum score (simulation 9). Expected Symptom Activity increases in comparison with baseline. This high-involvement profile, characterized by the presence in the network of high values of all strategies, was associated with increased network connectivity (more difficulties in emotion regulation).

#### Network Approach

Different network configurations among parent emotion socialization strategies are presented in simulations 10–32. In the first round of analyses, focusing on reward, reward was conditioned with their maximum score and override with their minimum score, and alternately conditioned the other three emotion socialization strategies (simulation 10–16). In simulations 10–12, the other three emotion socialization strategies were conditioned with their minimum score, conditioning one at a time with their maximum score. Expected Symptom Activity decreases in comparison with baseline, except for simulation 11 (reward and neglect maximum score), in which Expected Symptom Activity increases. High reward was associated with decreased network connectivity (less difficulties in emotion regulation) in the presence of low override and high punishment and magnify, but not high neglect. In simulations 13–15, one of the three emotion socialization strategies were conditioned with their minimum score and the other two with their maximum score, conditioning two of a time with their maximum score. Expected Symptom Activity increases in comparison with baseline. The high reward was associated with increased network connectivity (more difficulties in emotion regulation) in the presence of low override and when there is a combination of high values of two other emotion socialization strategies (punishment, neglect, or magnify). In simulation 16, the other three strategies were conditioned with their maximum score. Expected Symptom Activity increases in comparison with baseline. The high reward was associated with increased network connectivity (more difficulties in emotion regulation) in the presence of low override, and when there is a combination of high values of emotion socialization, the other emotion socialization strategies (punishment, neglect, and magnify).

In a second round of analyses, focusing on override, reward was conditioned with their minimum score and override with their maximum score, and alternately conditioned the other three emotion socialization strategies (simulation 17–23). In simulations 17–19, the other three emotion socialization strategies were conditioned with their minimum score, conditioning one of a time with their maximum score. Expected Symptom Activity increases in comparison with baseline. The high override was associated with increased network connectivity (more difficulties in emotion regulation) in the presence of low reward and high punishment, neglect, and magnify. In simulations 20–22, one of the other emotion socialization strategies was conditioned with their minimum score and the other two with their maximum score, conditioning two of a time with their maximum score. Expected Symptom Activity increases in comparison with baseline. The high override was associated with increased network connectivity (more difficulties in emotion regulation) in the presence of low reward and when there is a combination of high values of two other emotion socialization strategies (punishment, neglect, and magnify). In simulation 23, the other three strategies were conditioned with their maximum score. Expected Symptom Activity increases in comparison with baseline. The high override was associated with increased network connectivity (more difficulties in emotion regulation) in the presence of low reward, and when there is a combination of high values of emotion socialization, the other emotion socialization strategies (punishment, neglect, and magnify).

Third, reward and override were conditioned with their maximum score and alternately conditioned the other three emotion socialization strategies (simulation 24–29). In simulations 24–26, the other three emotion socialization strategies were conditioned with their minimum score, conditioning one of a time with their maximum score. Expected Symptom Activity decreases in comparison with baseline. High reward and high override were associated with decreased network connectivity (fewer difficulties in emotion regulation) in the presence of high punishment, neglect, and magnify. In simulations 27–29, two of the other emotion socialization strategies were conditioned with their maximum score and the other one with their minimum score. Expected Symptom Activity increases in comparison with baseline. High reward and high override were associated with increased network connectivity (more difficulties in emotion regulation) when there is a combination of high values of two other emotion socialization strategies (punishment, neglect, and magnify).

Finally, reward and override were conditioned with their minimum score and the other three emotion socialization strategies with their maximum score, conditioning one of a time with their minimum score (simulation 30–32). Expected Symptom Activity increases in comparison with baseline. Low reward and low override were associated with increased network connectivity (more difficulties in emotion regulation) when there is a combination of high values of two other emotion socialization strategies (punishment, neglect, and magnify).

## Discussion

Previous research on parental emotion socialization has analyzed these strategies in isolation, without considering their interdependence. As a result, the dynamics between strategies remain unclear, and inconsistencies persist regarding the supportive / unsupportive role of each one. This article used a network analysis approach, applying Expected Symptom Activity (Lunansky et al., 2021) to parental emotion socialization strategies to provide information about the connection of these strategies to adolescents’ emotion regulation difficulties. Emotion socialization strategies were analyzed within an interactive and dynamic pattern/model constituted by the co-occurring strategies and the outcomes with which they are related (emotion regulation). These results provide some evidence consistent with previous findings (e.g., the positive role of reward), as well as a new and detailed understanding of the relation between adolescents’ difficulties in emotion regulation and each paternal emotion socialization strategy in response to adolescent’s negative emotions in the context of co-occurring emotion socialization strategies.

Regarding the first aim, higher parental comfort and empathy (reward) of negative emotions were associated with decreased adolescents’ difficulties in emotion regulation when the other four strategies were minimally used. This is consistent with the stated adaptive role of reward on emotional development, frequently referred to as emotional coaching (Breaux et al., 2022). Regarding override, for which mixed results were identified in the literature (McNeil & Zeman, 2021), these results support that it may be an adaptive strategy since higher parental dismissing or distracting behaviors were also associated with decreased difficulties in emotion regulation, when the other four strategies were minimally used. It might be the case that adolescents perceive parental behaviors such as telling them to cheer up, not to worry and buying them gifts as comforting attitudes and not as silencing their emotional expression. This interpretation is consistent with previous research indicating that the impact of parental responses may depend on cultural context (Rapp et al., 2022). Although there has been an increasing shift toward on individualistic values (Prioste et al., 2017), Portugal retains many collectivist-oriented values, where family cohesion and emotional moderation are highly valued (Prioste et al., 2015). In this context, override behaviors may be perceived as supportive rather than dismissive. Similar results have been found in other collectivist contexts, for instance, Latina mothers were found to minimize their children’s negative emotion, without this being linked to negative child outcomes (Lugo-Candelas et al., 2015). These findings suggest that the perceived impact of override strategies may depend on cultural norms and adolescents’ interpretations, which could explain why override was linked to fewer difficulties in emotion regulation in this study.

On the other hand, higher parental disapproval (punishment), unavailability (neglect), and parental responses with the same emotions with equal or stronger intensity (magnify) were associated with increased levels of difficulties in emotion regulation, when the other four strategies were minimally used. This is consistent with previous findings stating their role as unsupportive parental reactions (e.g., Klimes-Dougan et al., 2007).

Concerning the second aim, when comfort (reward) and dismissing / distracting (override) strategies were both highly used by parents, and the other three strategies were minimally used, there were lower difficulties in emotion regulation. In opposition, when high levels of expressing disapproval (punishment), showing unavailability (neglect), and responding with the same emotions (magnify) were present, and the other two adaptive strategies were minimally used, emotion regulation difficulties increased. This is consistent with previous findings that showed that parents’ supportive profiles (coaching / accepting) were associated with children displaying more adaptive emotion regulation, and parents’ unsupportive profiles (punishing / minimizing) were associated with children displaying more maladaptive emotion regulation (Howe & Zimmer-Gembeck, 2022; McKee et al., 2022; Wang et al., 2019).

Consisting with a previous study, when parents minimally used all emotion socialization strategies an association with better emotion regulation was found (Howe & Zimmer-Gembeck, 2023). However, others point to a negative role of this kind of parental low-involvement profile (Howe & Zimmer-Gembeck, 2022; McKee et al., 2022; Miller et al., 2015; Wang et al., 2019). At first glance, this contradicts expectations, as previous literature normally states uninvolved parenting as neglectful (Goagoses et al., 2023). Uninvolved parents often do not provide essential structure and emotional support, leading to emotion regulation difficulties (Morris et al., 2017), increased risk for mental health problems, such as depression and anxiety, while also disrupting secure attachments (Aldao et al., 2016). However, it is possible that adolescents perceive a low-involvement profile in a positive way due to the egocentric characteristics of the adolescent developmental stage (Elkind, 1967; Moretti & Peled, 2004). In other words, adolescents often feel that they are the focus of everyone’s attention, while being more self-focused. In addition, it is normal for the amount of time spent with parents to decrease as peer interactions increase. Nevertheless, during this stage of development, the parent-child relationship is undergoing significant changes as children strive for autonomy and parents adapt their support to the context of a different relationship (Moretti & Peled, 2004). As a result, adolescents may perceive less parental involvement as a sign of respect for their privacy and autonomy rather than a lack of care. Another possible explanation is that parents may be perceived as consistent and predictable, using both supportive and unsupportive strategies. Their low use of all parental strategies allows adolescents to know what to expect. This explanation may be supported by these findings on high parental involvement patterns, as an association with increased difficulties in emotion regulation was found.

High parental involvement (high use of all emotion socialization strategies) might be perceived as inconsistent or unpredictable (i.e., high use of different and opposing strategies; Arikan & Kumru, 2023). Adolescents may perceive contradictions in their parents’ emotion socialization strategies when, for example, they alternate between reinforcing and punishing or ignoring the same emotional responses in different situations. While rewarding aims to validate emotional expression, punishing or ignoring seeks to suppress it. Adolescents may perceive high-involved parents as incoherent, sometimes highly supportive, and, at other times, highly unsupportive. Therefore, it seems that adolescents perceive low-involved parents as less problematic compared to high-involved parents.

Overall, the results from the first and second aims of this study, which generally test the association of a certain emotion socialization strategy on emotion dysregulation when the other emotion socialization strategies are scarcely used, substantiate reward and override as supportive strategies, while punishment, neglect, and magnification as unsupportive strategies. These findings, relating to the network-centered approach (third aim), offer insight into the implications of more complex combinations of emotion socialization strategies.

First, the interconnections with other emotion socialization strategies that may limit the positive association of the reward strategy were discussed. Since override also showed a positive impact on the network, its effect was partially out by setting it to its minimum value. Results showed that when higher parental comfort and empathy (reward) co-occur with parents’ highly expressed disapproval (punishment) or high response with the same emotion (magnify), there was no association with increased difficulties in emotion regulation. These results seem to demonstrate the protective role of parents’ reward strategy for adolescents’ emotion regulation, over and above the negative effect of parents using punishment or magnifying their emotional responses (if only one of these is present). The relevance of reward can be corroborated by the network centrality results, which showed that this emotion socialization strategy has a high capacity to influence other nodes in the network. Previous research using a network approach suggest that targeting nodes with higher capacity to influence the network may prevent the activation/deactivation of the network interactions between the other nodes, promoting a healthier state (Castro et al., 2019). This means that reward may be a good target for intervention.

This study demonstrated that using reward will impact the association of the negative strategies (punish and magnify) so that the combination of these parenting reactions results in better emotion regulation. This result goes beyond the well-documented positive role of reward (Breaux et al., 2022), studied individually. This design suggests that punishment and magnification, in the presence of high reward behaviors might be perceived by youth as exceptions to the rule, thereby diminishing their relevance and negative impact. In the context of parenting, punishment and magnification, which intend to reduce negative affect, coexist with behaviors that promote adolescent’s tolerance to negative emotions through comforting, empathizing, and problem-solving (reward). Alternatively, youth may sometimes perceive these purported negative behaviors as parental attempts to help them regulate emotions. For example, when parents discourage the expression of emotions (punish), this may result in helping the adolescent detach from their emotions state, that might be overwhelming, and allow them to initiate other emotion regulation strategies or problem-solving, as well as learn when emotional expression is inappropriate (O’Neal & Magai, 2005). The same may occur when high magnification is combined with high reward. Magnification could be perceived as an empathetic response since parents demonstrate the same emotions as their children (i.e., parents being sad when the adolescent is sad; Klimes-Dougan et al., 2007). When parents show the same emotions, they can also display how they regulate their own emotions, helping their children learn to regulate their emotions by modeling their parents’ emotion regulation strategies (Zimmer-Gembeck et al., 2019). As mentioned above, these results seem consistent with research on the impact of cultural context, suggesting that parents in collectivist-influenced cultures often display a combination of warmth and control when interacting with their children (Ispa et al., 2004). As such context, emotion socialization strategies that might be labeled as non-supportive could be interpreted as consistent with cultural values, and therefore supportive (Rapp et al., 2022). These findings also suggest that, in Portugues adolescents’, parental expression of disapproval of negative emotion, may have a less harmful effects, when reward is highly present.

On the other hand, when parents largely ignore their children’s emotions and are unavailable to them (neglect), the supportive role of higher parental comfort (reward) does not prevail, and there are increased emotion regulation difficulties. The negative importance of parental neglectful behaviors is also evident in the network centrality results, where neglect has the second highest capacity to influence another node in the network. These results seem contrary to one study that found that Latina mothers were more likely to neglect their children’s negative affect, yet this response was not associated with poor child outcomes (Lugo-Candelas et al., 2015). These findings may be related to the increasing changed toward individualistic values that emphasize children emotional expression, suggest that in Portugues adolescents’, parents ignoring their children’s negative emotions, may have more harmful effects. Therefore, neglectful parental reactions may also be preferential targets for intervention in Portugal and other cultures.

Adolescents’ perception of parental neglect seems to be more detrimental to their emotion regulation than punishment or magnification. Youth may perceive neglectful parental responses as emotional unavailability or lack of love, and these responses deprive them of the external resources necessary to manage, express, and regulate their emotions (Goagoses et al., 2023). This, in turn, leads to insecure attachment patterns (Moretti & Peled, 2004), use of maladaptive emotion regulation strategies, and increased risk for mental health problems (i.e., anxiety and depression symptoms; Glickman et al., 2021). Moreover, neglect contrasts profoundly with reward both in the behaviors expressed and in its association to adolescents’ emotional development. Parental reward reinforces secure attachment and fosters emotional and behavioral development (e.g., enhanced emotion regulation, self-esteem, and resilience) by providing emotional support through active listening, empathetic responses, and validation of their children’s emotions. Therefore, when parents alternate between highly rewarding and highly neglectful responses, conceptualized as opposite emotion socialization parental reactions, adolescents may perceive parents as contradictory and unpredictable. As mentioned above, parental inconsistency can create confusion for adolescents resulting in feelings of uncertain about how parents may react to their emotions, making it difficult for adolescents to develop stable and effective emotion regulation strategies.

Coming back to the analysis of the buffering impact of reward on adolescents’ emotion regulation difficulties, the presence of two or more unsupportive strategies extinguishes that positive effect (high punishment and neglect; high punishment and magnify; high neglect and magnify; or high punishment, neglect and magnify) since there is an association with increased adolescents’ emotion regulation difficulties. In these scenarios, probably fewer parent-youth interactions featuring reward behaviors will be present, and the negative consequences of the other parental practices, and the experience of inconsistency may be more frequent. Additionally, in the presence of two or more elevated purported maladaptive strategies, adolescents may no longer perceive unsupportive responses as attempts to help them regulate their emotions or exceptions to a characteristic supportive parental behavior. This result goes in line with the negative association of high-involved parents.

Second, the focus was on the interconnections of override with other emotion socialization strategies that may limit the positive association of the override strategy. As said before, override is associated with decreased difficulties in emotion regulation when parents scarcely use reward and the other three unsupportive strategies. For override to lose its protective effect, it only takes that parents also extensively use one unsupportive emotion socialization strategy. Compared to the buffering effect of reward, overriding is shown to be less effective. However, when rewarding behaviors co-occur with high override and neglectful behaviors, neglect no longer impact the network and emotion regulation difficulties decrease. This is a relevant finding because it shows how override responses may be supportive of youth emotion regulation development, but only in certain circumstances, and therefore, the importance of analyzing the co-occurring parental reactions.

This network approach, may therefore answer, at least partially, why there are mixed results found in the literature, pointing both ways for a positive and negative role of the override strategy (McNeil & Zeman, 2021). In this study, it seems that adolescents perceive parental distraction behavior positively. This may be due to the immediate relief, reduction of conflict, and engagement in enjoyable activities that override promotes. This approach can be perceived as supportive, helping adolescents avoid uncomfortable situations (Morris et al., 2017). Nonetheless, it seems important to balance distraction behaviors, that do not facilitate emotion awareness and understanding (McNeil & Zeman, 2021) with the development of emotional processing skills, facilitated by parental reward (Aldao et al., 2016; Morris et al., 2017). This balance seems to be observed in these results since combining reward with override enhances their individual positive impacts. These strategies seem to complement each other, promoting greater emotional resilience and more effective management of emotional challenges (McNeil & Zeman, 2021).

Furthermore, this study emphasizes that no parent-specific response is invariably beneficial or detrimental. Real-life parenting encompasses a variety of behaviors, emitted based on parental emotional resources, history, and cultural and contextual demands. The engagement in one unsupportive emotion socialization strategy does not necessarily harm adolescents’ emotion regulation. This shows the importance of analyzing the co-occurring parental reactions and implementing interventions that are more aligned with a realistic “good enough parenting” approach (Arikan & Kumru, 2023; Cirasola, 2024), that does not blame parents for not being perfect and that should target as high-priority objectives diminishing parental neglectful socialization practices and increase reward and probably override behaviors.

### Limitations and Future Directions

Several limitations are found in this study. First, it focuses exclusively on the adolescents’ standpoint, which introduces potential reporting bias, as their perceptions of parental reactions may differ from their parents’ perceptions. Indeed, the agreement between parent’ and adolescent’ reports is generally moderate to low, highlighting the importance of considering multiple informants (Achenbach, 2006). Although not collecting data from other informants or relying on other methods (e.g., observation) can be considered a limitation, focusing on youth’s perspective can also be viewed as an important aspect since parents’ perceptions receive more attention in research compared to their children’s (Azevedo et al., 2023). Future research should consider including both adolescent and parent as informants to assess whether significant differences emerge.

Second, this study focuses on a wide age range of participants (middle adolescence), when changes in the parent-adolescent relationship and youths’ emotion regulation capacities may be observed (Marceau et al., 2015). However, the sample size of this study would not allow for separate network analyses across different age groups without compromising the stability and robustness of the network analyses and also would lead to a massive number of results, making interpretation even more complex. Therefore, future research designs could accommodate this research question, that features further exploration.

Third, this study focuses on negative emotions without differentiating/separating emotions (e.g., sadness, anger, and fear). If this network analyses were to be applied to several emotions it would lead to cumbersome results, hard to interpret. Nevertheless, research shows that parents may have different socialization reactions to different emotions, and that emotion socialization strategies may contribute in unique ways, to the expression of that specific emotion in adolescents, to emotion regulation, and to psychological adjustment (Hu et al., 2023; O’Neal & Magai, 2005). Therefore, parental emotion socialization reaction to the different adolescents’ negative emotions demands further analyses.

Future research should focus on other important features of these analyses, such as interpreting the “effect size” of the differences in network activity. Moreover, mapping the interactions and their directionality between emotion socialization strategies and between these strategies and adolescents’ outcomes (i.e., specific emotion regulation strategies/difficulties) in more detail would be relevant as previous research found bidirectional effects between emotion socialization strategies and adolescent’s emotion dysregulation (Chronis-Tuscano et al., 2022), as well as with adolescents’ symptoms (Jones et al., 2024). However, Expected Symptom Activity is a statistical method that focuses on a network structure and simulates how changes in external variables influence node activation given the established network. Therefore, to the best of current understanding, this approach does not allow to test bidirectionality or reverse causality in the network, nor the use of statistical indexes to quantify the differences in Expected Symptom Activity values. Both limitations offer promising directions for future developments in network analysis and Expected Symptom Activity methods.

Finally, cultural beliefs shape how parents respond to their children’s emotions and how children perceive these interactions (Gottman et al., 1996). This raises the question of whether these findings apply specifically to the Portuguese culture or are generalizable to other collectivist cultures or even individualistic ones, since individualistic values have also been emerging in younger generations in Portugal (Prioste et al., 2017). Cross-cultural studies could better clarify the quality of the emotion socialization strategies (i.e., supportive / unsupportive role) across different cultures.

Nevertheless, taking into consideration the results of this investigation, future interventions should feature as a priority reducing neglectful behaviors and reallocating interaction time to strategies that help buffer their effects. These may include providing emotional support through active listening, empathic responses, and validation, as well as distracting behaviors such as encouragement or offering reassurance.

## Conclusion

Parental emotion socialization practices play an important role in how adolescents learn to express and regulate emotions, but there are inconsistencies in the literature regarding the supportive/unsupportive roles of each strategy. Addressing this gap, Expected Symptom Activity was applied to assess how specific parental emotion socialization strategies, in combination with others, related with adolescents emotion regulation difficulties. The findings showed that typically negative responses like discouraging emotional expression (punish) or mirroring their children’s emotional activation (magnify), may be less harmful when paired with supportive behaviors such as reward. In contrast, parental emotional unavailability and ignoring adolescents’ emotions emerged as more detrimental. By emphasizing the dynamic nature of parenting, this study contributed to a more detailed understanding of parental emotional socialization in middle adolescence, showing that positive emotion socialization responses can co-occur, to some extent, with negative ones and even so, be associated with less emotional regulation difficulties in youth, which is consistent with a “good enough” parenting perspective and finally, that neglectful parental behaviors should be targeted as a priority in interventions.

## Supplementary information

Supplementary Information
