# Supplementary material for: Parental Emotion Socialization and Difficulties in Emotion Regulation in Adolescents: A Network Analysis
Source: J Youth Adolesc. 2025 Jul 30;54(11):2778–93. doi: 10.1007/s10964-025-02222-8 (PMC12532742; doi:10.1007/s10964-025-02222-8)
Supplement: Supplementary file 1 — Supplementary Information [file 10964_2025_2222_MOESM1_ESM.docx]

Supplementary Materials

**Parental Emotion Socialization and Difficulties in Emotion Regulation in Adolescents: A Network Analysis**

**Explanation of the Network Analysis**

In this study, we used network analysis methodology. A network model represents a complex system composed of nodes and edges. Nodes represent the individual elements or variables in the system, while edges indicate the relationships or connections between those variables (Borsboom et al., 2021; Epskamp & Fried, 2018). This approach is used across various fields (e.g., physics and mathematics; Borsboom & Cramer, 2013). In psychology, a network model is often applied to understand mental disorders by mapping symptoms as nodes and the statistical relationships between them as edges (Fried et al., 2017). In the present study, a network model of the associations between emotion socialization strategies and difficulties in emotion regulation was estimated.

The workflow of network approaches involves three main steps, specifically, network structure estimation — applying statistical models to assess the structure of pairwise conditional associations in the data (i.e., pairwise Markov random field); network description — characterizing the global network topology and the centrality of nodes; and network stability analysis — evaluating the accuracy of edge weight estimation and robustness of the network to sampling error (Borsboom et al., 2021).

**Network Structure Estimation**

Given that cross-sectional data was used in this study, the network represents the conditional associations between variables at a single point in time. Accordingly, the network was estimated using a Mixed Graphical Model (MGM; Epskamp et al., 2018; Haslbeck & Waldorp, 2020). The package used to estimate the network was *bootnet* package in R with *mgm* default (version 1.5.5; Epskamp & Fried, 2023). Since all variables were continuous, edges are parameterized as partial correlation coefficients. The thickness of each edge indicates the strength of the conditional association between nodes (i.e., edge-weight). Model selection was performed using 10-fold cross-validation to select the regularization parameter, balancing the model fit and sparsity. No thresholding was applied.

**Network Description**

The network structure presented in Figure 1 (manuscript) consists of 11 nodes, organized into two theory-based communities: emotions socialization strategies (i.e., reward, override, punishment, neglect, and magnification) and difficulties in emotion regulation (i.e., limited access to emotion regulation strategies, non-acceptance of emotion responses, lack of emotion awareness, impulse control difficulties; difficulties acting in accordance with goals, and lack of emotion clarity). These communities are presented with different colors, facilitating the identification of variables that are closely related within and across groups. The *qgraph* R package (version 1.9.2; Epskamp et al., 2012, 2023) was used to visualize the estimated network.

Network density refers to the proportion of actual connections (edges) present in a network relative to the total number of possible connections. A higher dense network indicates a more fully connected network, whereas a sparse network suggests that fewer edges are presented relative to the total number of possible connections (Borsboom et al., 2021). This sparsity is typically achieved through regularization techniques that limit the inclusion of spurious edges (or false positives). A sparser network enhances both the interpretability and stability of the model by highlighting only the most robust associations (Epskamp & Fried, 2018). In the present study, the network density of the edges between nodes was .141, indicating that approximately 14.1*%* of all possible edges were retained. This reflects a moderately sparse network structure.

The edge-weights table (Table S1, supplementary materials) presents the estimated partial correlations between variables in the network. Most edges have small to moderate weights. Notably, some edges display stronger connections, such as the negative association between *reward* and *neglect* (-.776), and positive associations like those between *limited access to emotion regulation strategies* and *non-acceptance of emotion responses* (.462). Most edge-weights are close to zero, consistent with the overall network sparsity.

The network centrality analyses (Figure 2) focused on node strength, computed as the sum of the absolute values of edges connected to each node (i.e., partial correlation coefficients). Node strength was standardized using z-scores to assess the importance of each node within the network and their capacity to influence other nodes. This measure provides insight into which nodes are the most influence by being strongly connected to many other nodes (Epskamp et al., 2018). In this network, the nodes with the highest strength were *limited access to emotion regulation strategies* (1.60), *neglect* (1.27), and *reward* (1.22), indicating these variables were the most influential within the network (Table S2, supplementary materials).

**Network Stability Analysis**

To assess the reliability and robustness of the estimated network, it is important to evaluate the accuracy of its estimated parameters, such as edge strengths and node centrality (Epskamp et al., 2018). Therefore, a bootstrapping method was used to re-estimate the network through data resampling, allowing for the evaluation of the consistency of estimated parameters across samples (Epskamp et al., 2018). As proposed (Borsboom et al., 2021; Epskamp et al., 2018), four plots were used to assess the robustness and stability of the network (Figure S1, S2, and S3, supplementary materials).

**Edge-weight accuracy.** Edge-weight accuracy was evaluated using nonparametric bootstrapping resampling, which provided 95% confidence intervals around the estimated edge-weights (Figure S1, supplementary materials). Bootstrap results indicated that most edge-weights had relatively narrow confidence intervals, indicating satisfactory stability and supporting their reliable interpretation.

**Bootstrapped difference tests.** Bootstrapped difference tests were conducted to assess whether certain edges were significantly stronger than others (Figure S2a, supplementary materials) and whether nodes significantly differed in their overall strength (Figure S2b, supplementary materials). The results showed that several edge-weights were significantly different from each other, indicating important structural differences within the network. Additionally, differences in node strength centrality were observed, highlighting variability in the relative importance of nodes within the network.

**Centrality stability.** To assess the stability of centrality indices, a case-dropping bootstrap analyses was performed. This method evaluates how node strength remains consistent after systematically removing increasing proportions of the sample (Figure S3, supplementary materials). The results suggest moderate stability of strength centrality. The correlation stability coefficient (CS-coefficient) represents the maximum proportion of cases that can be dropped, such that with a 95% probability the correlation between the original centrality measures and the centrality measures based on the subset is .7. The CS-coefficient for the edge weights indicates good stability (CS(cor = 0.7) = .75), as does the CS-coefficient for the strength centrality (CS(cor = 0.7) = .52). Both values are above the recommended minimum threshold of .25 and even surpasses the preferably threshold of .50, indicating that the estimates remain robust even when a substantial portion of the sample is dropped (Borsboom et al., 2021; Epskamp et al., 2018).

Overall, the network stability analyses indicate the robustness of the network’s structure and centrality measures, providing confidence in the conclusions drawn from the model.

**Code availability**

R-scripts for these analyses, can be found here: https://osf.io/t2m5e/

**References**

Borsboom, D., & Cramer, A. O. J. (2013). Network analysis: An integrative approach to the structure of psychopathology. In *Annual Review of Clinical Psychology* (Vol. 9, pp. 91–121). https://doi.org/10.1146/annurev-clinpsy-050212-185608

Borsboom, D., Deserno, M. K., Rhemtulla, M., Epskamp, S., Fried, E. I., McNally, R. J., Robinaugh, D. J., Perugini, M., Dalege, J., Costantini, G., Isvoranu, A. M., Wysocki, A. C., van Borkulo, C. D., van Bork, R., & Waldorp, L. J. (2021). Network analysis of multivariate data in psychological science. In *Nature Reviews Methods Primers* (Vol. 1, Issue 1). Springer Nature. https://doi.org/10.1038/s43586-021-00055-w

Epskamp, S., Borsboom, D., & Fried, E. I. (2018). Estimating psychological networks and their accuracy: A tutorial paper. *Behavior Research Methods*, *50*(1), 195–212. https://doi.org/10.3758/s13428-017-0862-1

Epskamp, S., Costantini, G., Haslbeck, J., Isvoranu, A., Cramer, A. O. J., Waldorp, L. J., Schmittmann, V. D., & Borsboom, D. (2023). Package “qgraph” Graph Plotting Methods, Psychometric Data Visualization and Graphical Model Estimation. *Journal of Statistical Software*. https://doi.org/10.18637/jss.v048.i04

Epskamp, S., Cramer, A. O. J., Waldorp, L. J., Schmittmann, V. D., & Borsboom, D. (2012). Qgraph: Network visualizations of relationships in psychometric data. *Journal of Statistical Software*, *48*. https://doi.org/10.18637/jss.v048.i04

Epskamp, S., & Fried, E. I. (2023). *Package ‘bootnet.’* *50*(1), 195–212. https://doi.org/10.3758/s13428-017-0862-1

Epskamp, S., & Fried, E. (2018). A Tutorial on Regularized Partial Correlation Networks. *Psychological Methods*. https://doi.org/10.1037/met0000167.supp

Fried, E. I., van Borkulo, C. D., Cramer, A. O. J., Boschloo, L., Schoevers, R. A., & Borsboom, D. (2017). Mental disorders as networks of problems: a review of recent insights. In *Social Psychiatry and Psychiatric Epidemiology* (Vol. 52, Issue 1). Dr. Dietrich Steinkopff Verlag GmbH and Co. KG. https://doi.org/10.1007/s00127-016-1319-z

Haslbeck, J. M. B., & Waldorp, L. J. (2020). MGM: Estimating time-varying mixed graphical models in high-dimensional data. *Journal of Statistical Software*, *93*. https://doi.org/10.18637/jss.v093.i08

**Table S1**

*Weights of the Edges in the Emotion Socializations Strategies and Difficulties in Emotion Regulation Network*

|  | 1 | 2 | 3 | 4 | 5 | 6 | 7 | 8 | 9 | 10 | 11 |
| --- | --- | --- | --- | --- | --- | --- | --- | --- | --- | --- | --- |
| 1. RWD | - |  |  |  |  |  |  |  |  |  |  |
| 2. PNS | -.095 | - |  |  |  |  |  |  |  |  |  |
| 3. NGL | -.776 | .000 | - |  |  |  |  |  |  |  |  |
| 4. OVR | .401 | .178 | -.087 | - |  |  |  |  |  |  |  |
| 5. MAG | .000 | .370 | -.238 | .045 | - |  |  |  |  |  |  |
| 6. STR | -.088 | -.063 | .000 | .000 | .151 | - |  |  |  |  |  |
| 7. NNA | .000 | .161 | .000 | .014 | .000 | .462 | - |  |  |  |  |
| 8. AWR | .000 | .048 | .145 | -.015 | -.052 | .000 | -.072 | - |  |  |  |
| 9. IMP | .000 | .108 | .000 | .000 | .019 | .370 | .000 | .000 | - |  |  |
| 10. GOL | .000 | -.047 | -.067 | .026 | -.081 | .233 | .028 | .000 | .339 | - |  |
| 11. CLR | .000 | -.049 | .061 | -.058 | .129 | .086 | .126 | .437 | .086 | .065 | - |
| *Nota.* RWD: Reward; PNS: Punishment; NGL: Neglect; OVR: Override, MAG: Magnify; STR: Strategies; NNA: Nonacceptance; AWR: Awareness; IMP: Impulse; GOL Goals; CLR: Clarity. | | | | | | | | | | | |

**Table S2**

*Centrality Indices Values*

|  | Strength |
| --- | --- |
| Reward (RWD) | 1.215 |
| Punishment (PNS) | .215 |
| Neglect (NGL) | 1.269 |
| Override (OVR) | -1.016 |
| Magnify (MAG) | .065 |
| Strategies (STR) | 1.603 |
| Nonacceptance (NNA) | -.862 |
| Awareness (AWR) | -1.248 |
| Impulse (IMP) | -.610 |
| Goals (GOL) | -.755 |
| Clarity (CLR) | .123 |

**Figure S1**

*Edge-weight Accuracy*


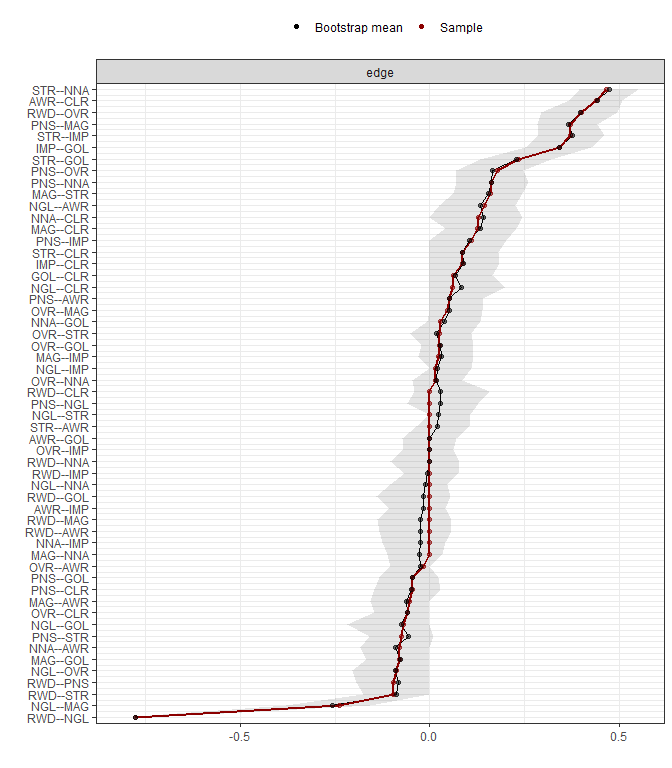


*Note.* Bootstrap confidence intervals were computed for the estimated edge-weights of the network. The red line indicates the sample values, the black line the average bootstrap value, and the gray area the bootstrapped 95% confidence intervals. RWD: Reward; PNS: Punishment; NGL: Neglect; OVR: Override, MAG: Magnify; STR: Strategies; NNA: Nonacceptance; AWR: Awareness; IMP: Impulse; GOL Goals; CLR: Clarity.

**Figure S2**

*Bootstrapped Difference Test*

a)

*
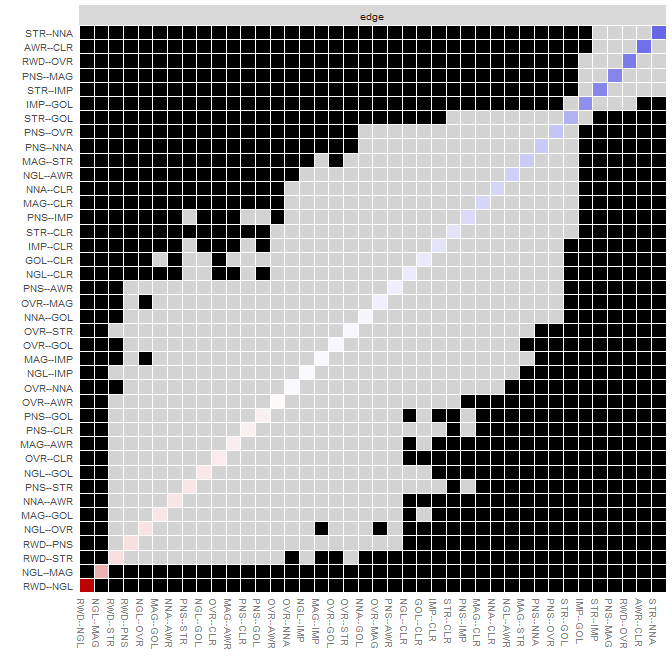
*

b)

*
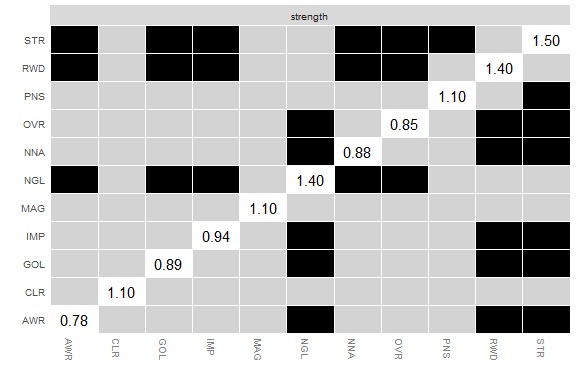
*

*Note.* Bootstrapped difference tests (*α* = 0.05) between non-zero- edges-weights (a) and the strength of the 11 nodes in the network (b). In both plots, the grey squares include the zero value, and the dark squares highlight the statistical significant difference, indicating whether two edges or two nodes are different from each other. In the above plot (a), diagonal represents magnitude of the edge, including the negative values (red color) and positive values (blue color). In the below plot (b), the white squares represent the value of node strength. RWD: Reward; PNS: Punishment; NGL: Neglect; OVR: Override, MAG: Magnify; STR: Strategies; NNA: Nonacceptance; AWR: Awareness; IMP: Impulse; GOL Goals; CLR: Clarity.

**Figure S3**

*Centrality Stability*

*
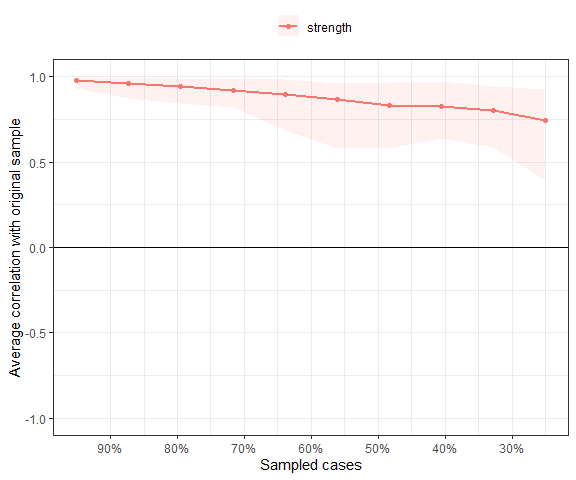
*

*Note.* Case dropping bootstrap analysis showing the average correlation between strength centrality indices of networks estimated sampled with case dropped (from 90% to 30%) and the original sample. The line indicates the average, while the shaded area indicates 95% bootstrapped confidence intervals of correlation estimates.
